# Policing, massive street drug testing and poly-substance use chaos in Georgia – a policy case study

**DOI:** 10.1186/s13011-016-0049-2

**Published:** 2016-01-16

**Authors:** David Otiashvili, Mzia Tabatadze, Nino Balanchivadze, Irma Kirtadze

**Affiliations:** Addiction Research Centre – Alternative Georgia, 14a Nutsubidze Str., Office 2, 0177 Tbilisi, Georgia; Business School, Ilia State University, Tbilisi, Georgia; National Centre for Commercial Law, Free University, Tbilisi, Georgia

**Keywords:** policing, drug testing, drug market, home-made drugs, poly-substance use, policy analysis

## Abstract

**Background:**

Since early 2000, intensive policing, wide scale street drug testing, and actions aimed at limiting the availability of specific drugs have been implemented in Georgia. Supporters of this approach argue that fear of drug testing and resulting punishment compels drug users to stop using and prevents youth from initiating drug use. It has been also stated that reduction in the availability of specific drugs should be seen as an indication of the overall success of counter-drug efforts. The aim of the current review is to describe the drug-related law enforcement response in Georgia and its impact on illicit drug consumption and drug-related harm.

**Method:**

We reviewed relevant literature that included peer-reviewed scientific articles, stand-alone research reports, annual drug situation reports, technical reports and program data. This was also supplemented by the review of relevant legislation and judicial practices for the twelve year period between 2002 and 2014.

**Results:**

Every episode of reduced availability of any “traditional” injection drug was followed by the discovery/introduction of a new injection preparation. The pattern of drug consumption was normally driven by users’ attempts to substitute their drug of choice through mixing together available alternative substances. Chaotic poly-substance use and extensive utilization of home-made injection drugs, prepared from toxic precursors, became common. Massive random street drug testing had little or no effect on the prevalence of problem drug use.

**Conclusions:**

Intensive harassment of drug users and exclusive focus on reducing the availability of specific drugs did not result in reduction of the prevalence of injecting drug use. Repressive response of Georgian anti-drug authorities relied heavily on consumer sanctions, which led to shifts in drug users’ behavior. In most cases, these shifts were associated with the introduction and use of new toxic preparations and subsequent harm to the physical and mental health of drug consumers.

## Background

The need for an evidence-based and balanced approach to illicit drug consumption has been repeatedly acknowledged in scientific literature and, increasingly, in political advocacy [1, 2]. With a growing number of jurisdictions willing to experiment with drug control approaches, the national and international debates on rational and effective policy models have been gaining momentum. An integral part of these debates has been focusing on the impact of different enforcement measures on illicit drug markets and patterns of drug use. While in the developed world there has been a fair amount of literature accumulated on the issue [3–6], research investigating this topic in low and low-middle income countries has been scarce. Current analysis aims to fill this gap through critical review of supply reduction interventions and associated changes in patterns of illicit drug use in the former Soviet Republic - Georgia.

Home of 3.7 million inhabitants, Georgia is situated in South Caucasus region and borders with Russian Federation (north), Azerbaijan and Armenia (east), Turkey (south) and the Black Sea (west). Following the fall of Soviet regime in 1991, Georgia went through a turbulent period of economic transformation and civil unrest. A perceived rapid increase in illicit substance use, in particular injection drug use has been attributed to the relaxation of social fetters and border control, severe economic crisis and reassessment of values [7, 8]. Neither the Georgian system of post-Soviet narcology (addiction medicine) nor the government were prepared to meet the challenges posed by escalation of drug use and associated problems [9]. Despite some moves towards the introduction of evidence-based health interventions (introducing methadone treatment in 2005) priority was given to repressive measures and supply control [10]. Since the mid-2000s, intensive policing, wide scale street drug testing, and measures aimed at limiting availability of specific drugs have been seen by the government as a solution to the country’s drug problem [11]. Annually, tens of thousands of people were detained in the streets and subjected to drug testing [12]. Positive tests resulted in an administrative fine (double of average monthly salary), if documented for the first time, or in criminal sanctions, including one-year imprisonment, if documented for the second and subsequent times during 12 months. In addition, popular campaigns demonising specific drugs, which were the most popular at the time, and law enforcement interventions targeting illicit supply of those drugs have been common. Supporters of this approach argued that fear of drug testing and following punishment compelled drug users to stop using, and prevented youth from initiating drug use. It was also stated that reduction in availability of specific drugs should be seen as an indication of the overall success of law enforcement measures [13]. The aim of the current review is to describe the drug-related law enforcement response in Georgia and to assess the impact of enforcement-based supply and demand reduction interventions on illicit drug consumption and drug-related harms.

## Methods

For this policy case study, we reviewed relevant literature that included peer-reviewed scientific articles, stand-alone research reports, country annual drug situation reports, technical programmatic reports and programme data. In addition, we made a detailed review of relevant national legislation and judicial practice for 2002–2014. The literature was obtained between March-May 2015 through searches of a number of databases and online resources. These included: academic databases (Medline, Scopus, Google Scholar), and online legal and statistical databases (Legislative Herald of Georgia, Parliament of Georgia, National Statistics Office of Georgia, Ministry of Justice of Georgia, Ministry of Internal Affairs of Georgia). Remarkable part of information was obtained through direct written requests to ministries and other government institutions (Ministry of Justice of Georgia, Ministry of Internal Affairs of Georgia, Supreme Court of Georgia, Office of Prosecutor General of Georgia, Ministry of Corrections of Georgia, Ministry of Labour, Health and Social Affairs of Georgia). Documents included in this review were those containing information on legislation, policy and framework documents, and law enforcement and public health statistics related to illicit drug use. Of particular interest were annual drug situation reports, research reports and other documents that focused on evidences and analysis of policy and law enforcement practice, and patterns of illicit drug use and other behaviours exercised by people who inject drugs (PWID).

Since there is no drug information system in Georgia that would make a comprehensive assessment of drug market changes, we relied on two major sources – results of two surveys that have been systematically collecting standardized and comparable data on current injection drug use (defined here as the last week or last month injection use). The first was a programme database of the Georgian Harm Reduction Network (GHRN) - a non-governmental non-profit organization that runs 14 low threshold programmes in 11 cities and is a single major provider of harm reduction services to PWID in Georgia. The Global Fund to Fight AIDS, Tuberculosis and Malaria (GFATM) has funded these services. Starting from 2007, GHRN has collected data on socio-demographics and injection practices (including drugs injected during last month) among current injection drug users utilizing its services. Respondents for this annual, brief (16 questions), paper based survey, administered by social workers at each site, were recruited based on a convenient sampling among the clients of needle exchange programmes in all 11 cities. Total sample size varied from 1,200 to 2,500 depending on a year of the survey. Data were entered into excel database and results of a descriptive analysis were reported by GHRN systematically. The second source was a bi-annual Bio-Behavioural Surveillance Survey (BBSS) that has been implemented since 2002 in major cities. Funding for BBSS survey was initially provided by the United States Agency for International Development (USAID) and last two waves were funded by GFATM. BBSS has been implemented by the consortium of public and private research agencies and has employed standardized methodology for all waves of the survey. This anonymous, paper-based, interviewer-administered survey, among other data, has been collecting information on current use (last week use for 2002, 2004, and 2006, and last month use for 2008, 2012, and 2014). The survey utilized Respondent Driven Sampling approach and recruited on average 1,600 current injection drug users for each wave in six largest cities of the country. BBSS reports, released at the end of each wave, included results of descriptive and bi- and multi-variate analysis. For the purpose of current review we summarized the results of descriptive analysis of GHRN and BBSS surveys. The primary outcomes of interest were prevalence and patterns of illicit drug use, measured through indicators of current use of four major injection drugs: heroin, buprenorphine, home-made ATS, and home-made opioids (desomorphine).

Our analyses included critical assessment of policy changes and law enforcement interventions along the timeline of events, and concurrent changes in patterns of use. While triangulating the data obtained from a number of sources, we considered relationships between enforcement and drug users’ behavior, examined the adaptations that drug injectors (and/or markets) made to enforcement measures and the resulting negative effects.

## Results

### Drug use patterns

**Fig. 1:**
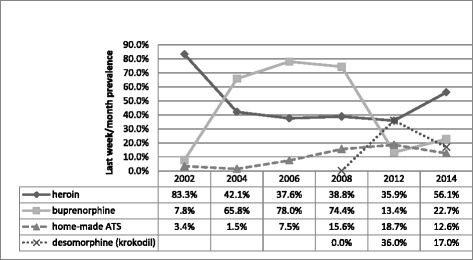
Trends in drugs injected in Georgia in 2002–2014 based on BBSS data (percentages exceed 100 % due to multiple responses, depending on a current poly-substance use by respondents)

In mid 1990s traditional raw opium was pushed out of the drug scene by rapid introduction of heroin, which was later partially replaced by buprenorphine in the form of Subutex® tablets [7]. Subutex® was reported as the primary drug of dependence for 40 % of patients admitted to inpatient treatment in 2005 [14]. About half of Subutex® injectors reported that they used it as a substitute for traditional opium or heroin [15]. At about the same time marked increase in injection use of home-made amphetamine-type stimulants (ATS) known as *vint* and *jeff* was observed (Fig. 1).

**Fig. 2:**
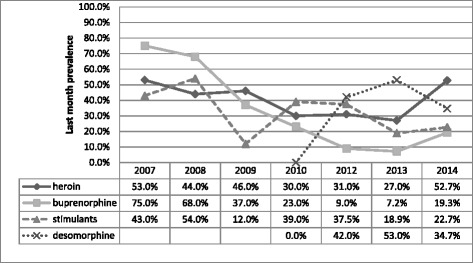
Trends in injecting drug use in Georgia 2007–2014 based on GHRN data (percentages exceed 100 % due to multiple responses, depending on a current poly-substance use by respondents)

The emergence of home-produced stimulants was evidently linked to increased mobility and labour migration to Russia and Ukraine, as well as diffusion of information, including recipes via internet [16]. By 2009 the prevalence of Subutex® injection use was dramatically reduced (from 75 % in 2007 to 7 % in 2013 - last month prevalence among drug injectors) [17]. By that time poly-substance use became widespread, with some studies reporting 90 % prevalence of concurrent use of two or more substances and 75 % prevalence of concurrent use of three and more substances among PWID [15]. Since then concurrent, often-unstructured use of multiple substances has remained an important characteristic of Georgian drug scene. For example, in 2014 on top of use of major injection drugs (as seen in Fig. 2) 42 %, 35 %, and 41.4 % of needle and syringe program (NSP) clients also reported use of alcohol, marijuana and sedatives respectively (data not shown).

In 2010 we documented first reports on the injection use of so-called “krokodil” (desomorphine), a home-made opioid produced from pharmaceutical drugs containing codeine [16]. By 2012 krokodil was the most frequently injected drug with 42 % and 36 % of drug injectors reporting last month use in GHRN and BBSS surveys respectively [18, 19] – see Figs. 1 and 2. Expectedly krokodil users composed a significant share of admissions in addiction clinics [20]. Prevalence of krokodil injection use was reduced from 53 % in 2013 to 34 % in 2014 (last month prevalence) in a GHRN sample, and from 36 % in 2012 to 17 % in 2014 in a sample of BBSS respondents. Both heroin and buprenorphine showed signs of increase, with the former drug regaining the status of the most often injected illicit substance for the first time since 2003 [21, 22].

To provide a full picture of the ever-changing drugs scene, it is important to mention the abuse of other products and over-the-counter medications that were popular for shorter periods of time and/or were characteristic to particular geographic areas of the country. Oral abuse of tramadol in combination with sedatives was reported in mid 1990s [7]. In 2003 Georgia experienced an explosive abuse of an injection preparation produced from poppy seeds that were normally used as an ingredient for different food (confectionery manufacturing) [7]. In 2005–2008 injection use of antidepressant tianeptin (Coaxil®) was widely reported from different regions, in particular west Georgia [23]. In 2010–2011 widespread oral consumption of anticonvulsant pregabalin (Lyrica®) was observed throughout the country [11]. And finally, in 2014 the first reports on injection use of eye drops Tropicamide® (benzeneacetamide) were released. Tropicamide® injections were reportedly used as a substitute by opioid injectors [17]. Table 1 presents the timeline and descriptive summary of events in relation to policy changes, drug use patterns and factors that contribute to changes in users’ behaviour.

**Table 1:** Timeline of major policy events and changes in drug use patterns

Timeline	Legal changes	Enforcement measures/practice	Drug use patterns	Factors influencing users behavior
Mid 1990s		• Widespread corruption among police • Drug users - subjects to harassment and extortion of bribes	• Opium↓ replaced by heroin↑ • Tramadol + sedatives↑	
Late 1990s	• Tramadol scheduled			
Early 2000s			• Heroin↓ replaced by buprenorphine (Subutex®)↑ • 40 % of treatment clients say Subutex® is primary drug • More than half report they use Subutex® to substitute opium or heroin • Home-made ATS (vint, jeff)↑	• Buprenorphine not detected by police urine testing • External signs of intoxication less visible • Increased mobility to Russia and Ukraine • Recipes via internet
2002–2003	• New Frame Law on Narcotics allowed substitution therapy • Poppy seeds scheduled		• Poppy seeds↑	• Price/availability
2005–2008			• Tianeptin (Coaxil®)↑ • Home-made ATS↑	• Price/availability • Not detected in police urine tests
2005	• Tianeptin scheduled	• First OST programme opened (GFATM)	• Subutex® and heroin - leading drugs	
2006	• War on crime - war on drugs • Adm. fine for drug use increased 5 fold • Drug testing facility moved to MIA	• Massive street drug testing launched • 12 fold increase in persons tested in 2007 vs 2006		
2007	• New law on Drug Crime (restrictions on civil rights)			
2007–2009		• Subutex-enemy #1 • Public campaigns “Anything but Subutex”, “Killer in the city”		
2008		• First state supported OST opened		
2009–2013		• Victory over Subutex® and heroin announced	• Subutex®↓last month use from 75 % in 2007 to 7 % in 2013) • Heroin↓ • Poly-subs use↑ (90 % of injectors use 2 or more drugs, 75 % use 3 or more drugs)	• Availability (police claimed they successfully collaborated with French counterparts to restrict smuggling of buprenorphine)
2009			• PDU size estimation - 40,000	
2010	• Pregabalin scheduled	• State funded OST scaled up - 17 sites • 4,600 patients treated in 2013 • Testing↓, imprisonment↓	• Pregabalin (Lirica®)↑	• Substitute for injectable opioids • Not in narcotics list • Not detected by police tests
			• Desomorphine (krokodil)↑	• Availability • Price • No need to engage with dealer/illicit market
2011	• Presidential Decree- Interagency Coordinating Council established			
2012			• Krokodil - leading inj. drug • PDU size estimation - 45,000	
2013	• National (anti)Drug. Strategy and Action Plan 2013–2015 • New Government came to power • Codeine tablets moved under strict control	• 60,000 tested • Testing↑, Imprisonment↑ • Krokodil named as most dangerous drug		
2014	• Law on New Psychoactive Drugs	• Victory over krokodil announced • MIA reported 90 % reduction in Krok. use (based on seizures and urine tests) • MIA reported 90 % reduction in NPS use (based on seizures and urine tests)	• Krokodil ↓( from 53 % in 2013 to 34 % in 2014, GHRN) • Heroin ↑ (heroin driven commuting to Turkey) • Buprenorphine ↑ • Tropicamide injection ↑	• Attempts to compensate for reduced desomorphine
2015			• PDU size estimation - 49,000	

### Policy interventions and legal practice

**Fig. 3:**
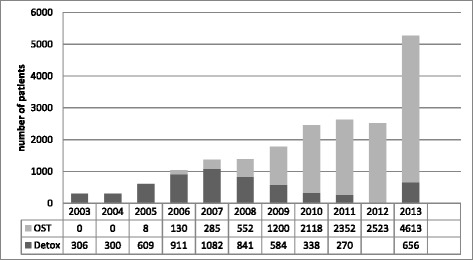
Number of patients treated for substance use disorders in 2003–2013. Fig. 6 in Alavidze, S., Balanchivadze, N., Batselashvili, L., Duchidze, N., Javakhishvili, J., Kikvidze, T., . . . Tsertsvadze, V. (2014). Drug Situation in Georgia 2013. In J. Javakhishvili, Otiashvili, D., Tabatadze, M. (Ed.). Tbilisi. Used with permission

We identified two major events that contributed to the improved availability of and access to evidence based treatment for substance use disorders. The first was the new Law on Narcotic Drugs, Psychotropic Substances, Precursors and Narcological Aid adopted in 2002 and which permitted use of substitution treatment with opioid agonists [24]. The treatment itself was launched in 2005 with the support from the GFATM. The second event was the decision of the Ministry of Labour, Health and Social Affairs to allocate funding for opioid substitution treatment and the launch of state co-funded programs in 2008. State support allowed for rapid expansion of the treatment. By the end of 2013, there were seven-teen treatment sites in the country that served a total of 4,600 patients annually [17] – see Fig. 3. Treatment of more than two thirds of those patients was co-funded by the state.

In relation to enforcement measures we distinguished three principal elements that were characteristic for drug-related response in last decade. The first was introduction of stricter measures over the control of specific substances in response to the increased abuse of these substances. In most cases this was done through moving the substance to another (more tightly regulated) list/schedule of controlled substances. This was done in relation to tramadol, poppy seeds, buprenorphine, tianeptin, pregabalin, codeine and few other substances.

Another element of Georgian legal response to drugs problem was introduction of legislation that would impose additional (to existing) punishment for drug related offences and/or new restrictions for civil rights of individuals engaged in illicit drug use. For example the New Law on Drug Crime adopted in 2007 introduced further restrictions for individuals sentenced for drug-related crimes. The law was intended to discourage illicit drug use via deprivation of driving license, arms license, a ban on passive election right, a ban on certain professional activities, (lawyer, physician, teacher and the like) and other rights [25].

**Fig. 4:**
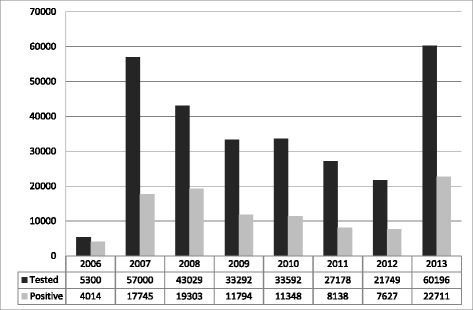
Number of individuals tested for drugs and number of positive results, 2006–2013. Figure 17 in Alavidze, S., Balanchivadze, N., Batselashvili, L., Duchidze, N., Javakhishvili, J., Kikvidze, T., . . . Tsertsvadze, V. (2014). Drug Situation in Georgia 2013. In J. Javakhishvili, Otiashvili, D., Tabatadze, M. (Ed.). Tbilisi. Used with permission

And finally, the critical element of the enforcement was large-scale street drug testing introduced since 2007. In late 2006 the Georgian government announced a war against crime, with drugs being one of the highest priorities. Massive drug testing was launched with a 12-fold increase in the number of persons tested annually between 2006 and 2007 [12]. The fine for the first time drug use (positive urine test) increased 5-fold and reached double the amount of the average monthly salary in the country [26]. Since then, annually, tens of thousands of people have been tested for presence of derivates of controlled substances in their urine (Fig. 4). Following the peak in 2007, the number of tested individuals was gradually decreasing until the new peak in 2013. Notably, the proportion of negative versus positive test results has remained stable – 2:1.

All these efforts were accompanied by wide scale TV public campaigns - “Killer in the city”, “Anything but Subutex®”, “Let’s defeat dependence together” – that supposedly aimed at preventing use of specific drugs (one per campaign), and focused on horrifying messages about immediate and inevitable harmful effects of those drugs (dependence, cancer, psychosis, death and so on) [11].

In an attempt to establish coordination mechanism for drug related activities the Inter-Agency Coordinating Council to Combat Drug Abuse was established by decree of the President. The Council was mandated to elaborate and coordinate the implementation of the national drug strategy and action plan [27]. In 2013 with the new government in power, the National (anti)Drug Strategy and Action Plan were developed and approved by the Interagency Coordinating Council. However, clear mechanisms of implementation and monitoring have not been established [17]. In the same year, a new wave of massive street drug testing was launched with a record of 60,196 episodes of drug testing performed (Fig. 4). In contrast, there were 5,269 individuals admitted for treatment (both drug free detoxification and opioid agonist maintenance treatment) for substance use related problems in the same year (Fig. 3).

## Discussion

The drug control regime in Georgia has been focusing primarily on law enforcement measures to target supply and availability of particular drugs and to reduce demand through imposing harsh punishment on drug users. There is a lack of evidence available suggesting any positive results in terms of reduction in the prevalence of problem drug use and/or its negative consequences. Reactive in its nature, the simplistic, repressive response heavily relied on consumer sanctions and stimulated shifts in drug markets and users behavior. In most cases these shifts were associated with the introduction and use of new toxic preparations and subsequent harm to the physical and mental health of individuals.

### Old markets limited - new markets explored

Efforts to limit the availability of particular drugs almost uniformly lead to an increase in use of other drugs, development of new sources of supply and/or production of new substances [28]. The heroin shortage in Australia in 2000–2001 provided a useful example of drug market shock in which the abrupt reduction in supply of heroin, with consequent increase in its price and decrease in purity and availability, resulted in a clear reduction in heroin use and increase in use of other drugs. The majority of heroin users reported a compensatory increase in consumption of cocaine, cannabis, benzodiazepines and methamphetamines [3, 29–31]. Another example was the spread of small-scale manufacturing of methamphetamine-like preparations utilizing over the counter cold medications and other more easily obtained chemicals, in response to the US government’s attempt to halt the illegal manufacturing of methamphetamine by regulating the sale and distribution of the precursor chemicals in the 1980’s. As stated by Cunningham and Liu, “the end result was an explosion in rural manufacturing and dangerous explosions of small kitchen labs used by meth addicts to cook up their own supplies of speed” [32].

Where illicit drug markets and distribution schemes are concerned, both traditional and novel control measures have been met with rapid countermeasures and technological innovations [33–35]. Recently, the rapid increase in use of new psychoactive substances (NPS) has brought a global change in drug markets, with the number of NPS already exceeding the number of psychoactive substances controlled at the international level [36, 37]. More than this, the Internet has shown to make an impact on the drug markets dramatically and has allowed information on drug use and production to spread rapidly, effectively facilitating the diffusion of new trends [6, 38].

Consistent with these findings, our research suggests that Georgian drug markets and drug users have shown a considerable capacity and innovation to adapt to new regulatory measures and increased scrutiny by law enforcement. Reduction in availability of traditional drugs led to the exploration and rapid growth in use of new drugs, mostly domestically manufactured substances. Describing and understanding organized drug distribution networks falls beyond the scope of this report. However, available data might suggest that these networks have been remarkably overshadowed by the rapid emergence of freelance distribution of Subutex® in mid-2000s (smuggling from Europe by leisure or business travellers [14]), as well as by user-driven kitchen production of injection preparations. It is to be investigated to what extent the Internet (for example, online recipes for self-production) has been influencing the development of Georgian drug markets.

This policy review provides unique example of a small country in which multiple specific policy interventions resulted in prompt and dramatic changes in drug consumption patterns. The scale and dynamics of these changes were impressive. Within 1–2 years, introduction and spread of new injection drugs/preparations, and a similarly rapid decline in consumption of these preparations evidently in response to targeted enforcement measures, only to give way to new substances. As argued in the following sections, the ultimate result of these changes did not seem to have led to any improvement in individual or public health. Rather, vice versa. Drug users switched to more toxic preparations and exercised more risky behaviour. However, the sensitivity and responsiveness of market players, seemingly very effective and rapid diffusion of information and new trends in the Georgian drug user setting can, and should, provide a window of opportunity (and become a focus of future research endeavours) for innovative approaches to educate and support behavioural changes aiming at reduction of negative consequences of substance use.

### Drivers behind changes in patterns of drug consumption

Development in substance use patterns is driven by a complex set of factors and socio-economic context. In many cases, drug use patterns in Georgia were largely shaped by policy response and law enforcement practices implemented at particular periods of time. Among others, these changes were driven by both consumers’ attempts to substitute the traditional drug of their choice and to make drug use less visible, so that risk of arrest would be reduced. In our previous report we suggested that less visible external signs of intoxication and absence of buprenorphine in the police drug testing kits played an important role in the rapid spread of Subutex® consumption in mid-2000s [15]. This lack of detectability obviously contributed to the spread of home-made ATS injection as well. As in case with buprenorphine, standard urine testing kits used by police did not include amphetamine and methamphetamine (at least at the initial stages), thus allowing users of *vint* and *jeff* to pass testing undetected. The same goes for other experimental drugs like Coaxil®, Lyrica®, and Tropicamid®. In addition, the risk of arrest was reduced since all the ingredients for kitchen production were obtained via pharmacy and convenience stores and there was no need to engage with drug dealers. Importantly, new injection preparations were remarkably cheaper - $5-7 per single dose of *vint*, *jeff* or krokodil, compared to $50-100 per single dose of heroin or buprenorphine.

Apart from directly influencing the supply of illicit drugs, law enforcement seeks to reduce the demand by making drugs expensive, hard to obtain and/or too risky to engage with. In Georgia politicians have argued that law enforcement (massive drug testing) reduces the demand by increasing the probability to get punished and thus coerces drug users to stop using drugs. In reality, the risk of detection is fairly limited and the improvement of detection rates is unrealistically expensive [28]. In the United States, cannabis users had a tiny 1 in 3,000 risk of being arrested for any given incident of cannabis use [4]. Simple calculations (45,000 problem injection users; roughly one injection per day; 20,000 positive results of the rapid urine toxicological testing in 2013 – see Fig. 4) give us about 1 in 1,000 risk of being arrested for any given incidence of injection drug use in Georgia. To be more accurate, this risk is even lower since 20,000 positive test results include non-injection/non-problem drug use as well, mainly marijuana. Obviously, this risk is low enough to support the assumption that it should drive the drug user’s decision to stop using. However low the risks of detection and arrest may be, it seems that Georgian drug consumers still did not ignore those risks. We believe that severe and grossly disproportionate punishments for possession/use of illicit substances involving long-term incarceration were important contributors to that. For example, possession of heroin in the amount of more than 1 gram, regardless of the purpose, is punished with 8 to 20 years of imprisonment or lifetime term (Art. 260 of the Penal Code of Georgia (III)). According to the same code, rape is punished with 4–6 years of imprisonment (Art. 137 of the Penal Code of Georgia) and murder is punished with 7–15 years in prison (Article 108 of the Penal Code of Georgia) [39]. Such harsh measures combined with large and frequent police engagement with drug users dramatically reduced the public visibility of people “under the influence” of psychoactive substances, driving drug users further underground and making their engagement with prevention and treatment services extremely difficult.

### New drugs – new harms

In an Australian study that described an increase in consumption of cocaine and other stimulants as a response to heroin shortage, Topp and co-authors suggested that “given the differential harms associated with the use of stimulants and opiates, this possibility has grave implications for Australia, where the intervention and treatment system is designed primarily to accommodate opiate use and dependence” [29]. On a positive note, reduction in overdose deaths and a possible reduction in injection drug use and hepatitis C infections were suggested to be potential public health benefits as a result of the Australian heroin shortage [3]. Switching to new drugs in Georgia was in many cases associated with increased risks for blood-borne infections and other harms often related to the toxic nature of ingredients used for preparation of self-manufactured injection solutions. Buprenorphine, home-made stimulants and home-made opioids all were obtained, processed and used in a way that required a group of injectors to collaborate. This was related to either collection of money for expensive 8 mg buprenorphine tablet, to be later divided and injected by, usually, four individuals, or to predetermined division of functions/labor (money, procurement of precursors, cooking) among group members in case of preparation of home-made stimulants and opioids [11]. In all these instances injection happened within a group of 3–5 drug injectors with apparently little direct sharing, but frequent indirect sharing via common container, cotton filters, and large volume syringe for division (front or back loading) of the produced substance [9, 40].

Addiction clinics and harm reduction programs reported numerous physical, neurological and psychiatric complications among consumers of home-made preparations, both stimulants and opioids, which were apparently linked to the toxicity of precursors used for processing – phosphorus, iodine, potassium permanganate, gasoline, strong acids and so on [41]. Soft tissue damages, necrosis, gangrene, osteomalacia and other severe impairments have been reported elsewhere in connection to injection use of krokodil and self-produced ATS [42–44]. Harm reduction services in Georgia have also reported increased demand for naloxone among krokodil injectors. Elevated risk of overdose was apparently associated with difficulties in titration and fluctuating quality/potency of self-manufactured krokodil [11].

Attempts to self-medicate and substitute traditional drugs of abuse have resulted in majority of drug injectors switching to unstructured poly-substance use. Dependent drug users consumed whatever intoxicants were available at the moment, often in combination, aiming to mimic their drug of choice and/or to increase the potency and prolong effects. Nine out of ten respondents in one survey (clients of needle and syringe exchange programmes) reported injecting at least two drugs, and two-thirds reported injecting three or more drugs during the last month [15]. This has apparently resulted in the rise in the number of overdose since most overdoses occur among individuals who consume multiple substances [45]. However, overdose cases are not properly documented in Georgia and we are lacking the data to support this assumption.

### Legal and ethical aspects of massive street drug testing

In Georgia, tens of thousands of people are subject to administrative and criminal proceedings (including sentencing to prison terms) as a consequence of positive rapid immunoassay test results. To the best of our knowledge, no other jurisdiction uses the results of rapid screening as irrecusably final evidence of drug use because of the issues related to the often-low specificity of the tests, cross-reactivity, and the stability of these devices (their ability to resist certain conditions, such as temperature and humidity). Elsewhere, these results are considered preliminary and indicative, and advanced confirmatory laboratory tests are required for legal proceedings both in criminal justice and workplace settings [46–49]. In Georgia, the results of these rapid tests are used as one single source of evidence in court, leading to heavy fines or imprisonment. We believe that this practice contradicts established international standards for a fair trial where sufficient evidence should be required for conviction beyond reasonable doubt of proof [50]. However, it is obvious that confirmatory testing of those who tested positive using initial on-site tests would increase the cost and expenditures of the testing intervention immensely.

We estimated that 1 in 20 men residing in the country was tested for drugs in 2013 (60,000 testing episodes among 1.4 mil men aged 15–64 [51] leaving in Georgia). Punitive measures, including massive street drug testing, that have no analogue in developed countries did not result in any measurable reduction in drug use in Georgia. Instead, such measures caused the harmful criminalization of thousands of otherwise law-abiding individuals. Importantly, these punitive measures had little or no influence on individual decisions to cease or to continue using drugs. Our earlier report showed that 89 % of individuals punished for drug use (as per positive drug test) returned to drugs immediately after the punishment, and 11 % did so within few months following the penalty [12]. Punishment did result in a change in user behaviour – study participants reported adapting variety of strategies to avoid being identified by police (injecting alone, injecting and staying at home, avoiding traveling by taxi and so on).

**Fig. 5:**
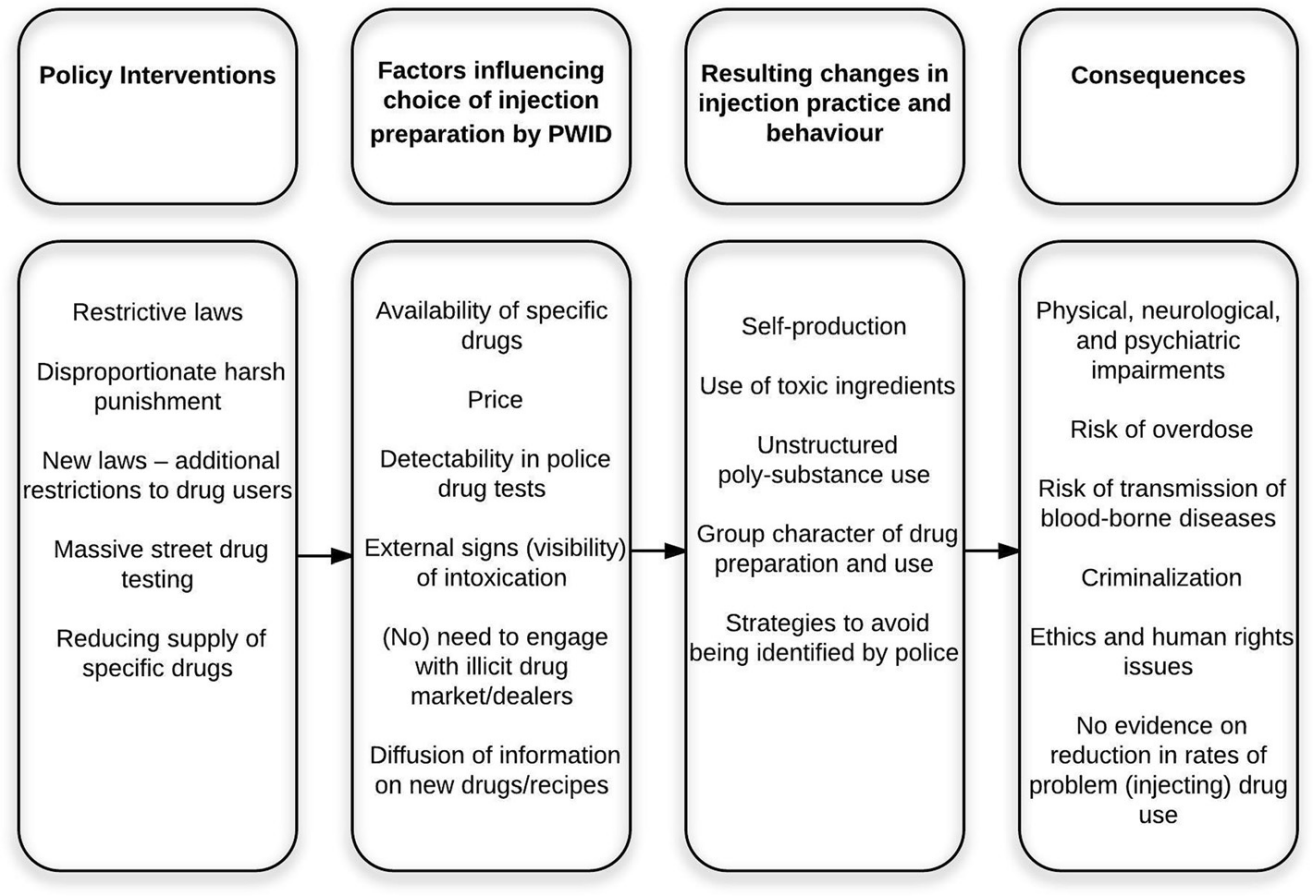
Policy interventions, evolving drug scene and implications

Finally, it is reasonable to state that massive drug testing, with the majority of the test results being negative, raises an ethical question. Subjecting tens of thousands of people to humiliating and lengthy drug-testing procedure infringes upon the dignity of citizens and undermines the public perception of a just and sound policy. Figure 5 presents a schematic description of drug policy interventions and relevant health and social implications in Georgia.

### Limitations

As with any research, our review has limitations.

#### Literature review

In our analysis we focused on associations between specific law enforcement interventions and changes in illicit drug markets and substance use patterns. However, shifts in drug trends are obviously not only a function of enforcement. Drug markets are responsive to different political, socio-economic and cultural forces. Nevertheless, analysing those factors, whatever the importance, was beyond the scope of current review. Secondly, due to lack or complete absence of data, we were unable to assess the effect of drug enforcement measures on initiation of drug use by new users.

#### Secondary analysis of drug use data

We believe that the reliability of drug use data analysed for this report was satisfactory. Differences in the prevalence of current use of specific drugs in two samples might suggest that these were somehow different drug using populations with distinct characteristics and behaviours. It is hard to estimate to what extend samples for two surveys overlap. Between 3.5 % (in Tbilisi) and 44 % (in Gori) of BBSS respondents reported receiving services from needle-syringe programs. Importantly, both surveys reported very similar trends in injection drug use over the years, which might suggest that these data correctly reflect availability and/or preferability of particular injection drugs by significant portion of Georgian PWID at specific periods of time.

Our general concerns relate to the overall fragmented character and limited scope of the data available in the country. Absence of comprehensive drug monitoring system in Georgia that would provide valid, relevant and continuous data was the major limitation. In order to measure changes in drug consumption, we focused on four major injection substances and relied on data provided by GHRN annual client survey and periodic BBSS. Supposedly, both surveys deal with problematic drug users, whom of which do not necessarily represent the entire substance-using population in the country. These problematic poly-substance users can respond to changes in a specific way, simply saying – consume whatever is available and rapidly switch to new drugs. Other groups (recreational, experimental users) may have responded differently to the changes in the legal environment, but there are no data to explore that. For example, as is the case with other countries [36], it is possible that new psychoactive substances, in fact, attracted new cohorts of users and they exercised responses and behaviours different from those of systematic injection users of drugs. Again, given the data available, we were unable to examine trends in NPS consumption and intentionally focused on four most prevalent injection drugs. Furthermore, since treatment options in Georgia are fairly limited and often subject to patient’s ability to cover the cost of the service, it was hard to measure whether reduction in supply/availability of specific drugs correlated with increased utilization of treatment. This is particularly true for prohibitively expensive drug free treatment and, to a lesser extent, to more affordable opioid substitution treatment. It is hard to suggest whether the visible increase in treatment episodes was a function of raising demand, or if it only reflected the persistently unsatisfied high demand for substance use treatment in the context of inadequate (maximum 10 %) coverage.

Finally, due to the lack of data, we did not discuss the price and purity of illicit drugs and did not cover the numbers of negative health and social consequences, including overdose death, morbidity due to toxic nature of home-produced injection preparations, criminal justice costs, social marginalization of users and their families. Some economic implications of massive street drug testing were reported earlier [12].

## Conclusions

This study highlights the need to re-examine national drug policy in Georgia. Intensive harassment of drug users and exclusive focus on reducing availability of specific drug(s), with no adequate emphases on health interventions, resulted in drug injectors in Georgia exploring new substances and switching to unstructured poly-substance use. The toxicity of new alternatives (home-made injection preparations), chaotic nature of mixing different substances, and the group character of consumption could be associated with a number of negative consequences and increased individual and public health risks.

Development in the drug scene and correlating drug markets is a dynamic process that requires thoughtful monitoring. Continuous documentation and analysis of policy interventions and subsequent changes in patterns of drug use and associated consequences is warranted in order to inform decision makers and allow for the formulation of rationale and effective policy responses. There is a need to establish a comprehensive drug monitoring system in Georgia that would provide professional community and policy makers with reliable, valid and systematic data on drug markets and drug use trends and patterns.

## Abbreviations

ATS: amphetamine-type stimulants
BBSS: Bio-Behavioral Surveillance Survey
Detox: drug free detoxification treatment
GHRN: Georgian Harm Reduction Network
MIA: Ministry of Internal Affairs
MOLHSA: Ministry of Labour, Health and Social Affairs
NSP: needle and syringe programs
OST: opiate substitution treatment
PDU: problem drug users
PWID: people who inject drugs
